# Effect of Edge-Oxidized Graphene Oxide (EOGO) on Fly Ash Geopolymer

**DOI:** 10.3390/ma18153457

**Published:** 2025-07-23

**Authors:** Hoyoung Lee, Junwoo Shin, Byoung Hooi Cho, Boo Hyun Nam

**Affiliations:** 1 Department of Civil Engineering, College of Engineering, Kyung Hee University, Yongin 17104, Republic of Korea; hoyounglee@khu.ac.kr (H.L.); bcho@khu.ac.kr (B.H.C.); 2 Department of Civil Engineering, Kumoh National Institute of Technology, Gumi 39177, Republic of Korea; shinjw@kumoh.ac.kr

**Keywords:** Geopolymer, Fly ash, EOGO, compressive strength, FFRC

## Abstract

In this study, edge-oxidized graphene oxide (EOGO) was used as an additive in fly ash (FA) geopolymer paste. The effect of EOGO on the properties of the fly ash geopolymer was investigated. EOGO was added to the FA geopolymer at four different percentages (0%, 0.1%, 0.5% and 1%), and the mixture was cured under two different conditions: room curing (~20 °C) and heat curing (~60 °C). To characterize the FA-EOGO geopolymer, multiple laboratory tests were employed, including compressive strength, Free-Free Resonance Column (FFRC), density, water absorption, and setting tests. The FFRC test was used to evaluate the stiffness at small strain (Young’s modulus) via the resonance of the specimen. The mechanical test results showed that the strength and elastic modulus were high during heat curing, and the highest compressive strength and elastic modulus were achieved at 0.1% EOGO. In the physical test, 0.1% EOGO had the highest density and the lowest porosity and water absorption. As a result of the setting time test, as the EOGO content increased, the setting time was shortened. It is concluded that the optimum proportion of EOGO is 0.1% in FA geopolymer paste.

## 1. Introduction

The global construction industry has grown based on the large-scale use of cement due to rapid urbanization and infrastructure expansion. However, this growth has also had negative effects on the environment. In particular, the carbon dioxide emissions generated during the manufacturing process of ordinary Portland cement (OPC) account for about 8% of global emissions, posing a serious environmental problem. Accordingly, there is an increasing interest in the development of alternatives that can reduce environmental impact while maintaining mechanical performance [1,2].

Geopolymers are one of the good alternatives to cement, made from materials rich in silica and alumina (such as fly ash and blast furnace slag). Recently, as the demand for thermal power plants has decreased, the supply of fly ash and slag has also decreased, and research is actively being conducted to find new aluminosilicate raw materials. Xiao et al. also proposed the application of waste glass powder as a new geopolymer material due to its abundant silica and stable chemical composition [3]. These materials do not require high temperature for calcination at the level of cement production, which offers advantages such as reduced carbon dioxide emissions and cost savings. Geopolymers are hardened through a polymerization reaction that forms a three-dimensional structure of aluminosilicate gel (N-A-S-H) in alkaline conditions [4,5,6,7]. This reaction mechanism has advantages such as early strength development compared to OPC, chemical resistance, heat resistance, and fast setting time [8,9,10,11].

However, fly ash-based geopolymers can experience issues such as crack formation and reduced durability due to their low reactivity at room temperature (23 °C) and the heterogeneity of the gel structure. To address these limitations, there have been recent active efforts to incorporate nanomaterials [12,13].

Research is being conducted to improve the performance and durability of geopolymers using nanomaterials, and nanomaterials such as nanosilica, nanoclay, and graphene are commonly used. Addition of less than 1 wt% of nanomaterial can improve mechanical properties such as compressive strength, flexural strength, and toughness, as well as affecting properties such as permeability, moisture adsorption, porosity, and thermal properties. Among these nanomaterials, graphene oxide (GO) stands out due to its high specific surface area, mechanical strength, electrical conductivity, and exceptional reactivity through surface oxygen functional groups (-OH, -COOH). In particular, GO can be expected to have several effects, including promoting gel formation in geopolymers, reducing porosity, and enhancing strength with only a small addition (less than 1 wt%). Several studies have reported that GO effectively improves the mechanical performance of geopolymers. In addition, GO is considered an effective additive because the structure of GO and the addition of oxygen groups can reduce the penetration of chloride ions and thermal conductivity [14,15,16,17]. However, the existing GO is used only to a limited extent as an admixture in construction materials due to its high cost relative to its exceptional performance.

**Figure 1:**
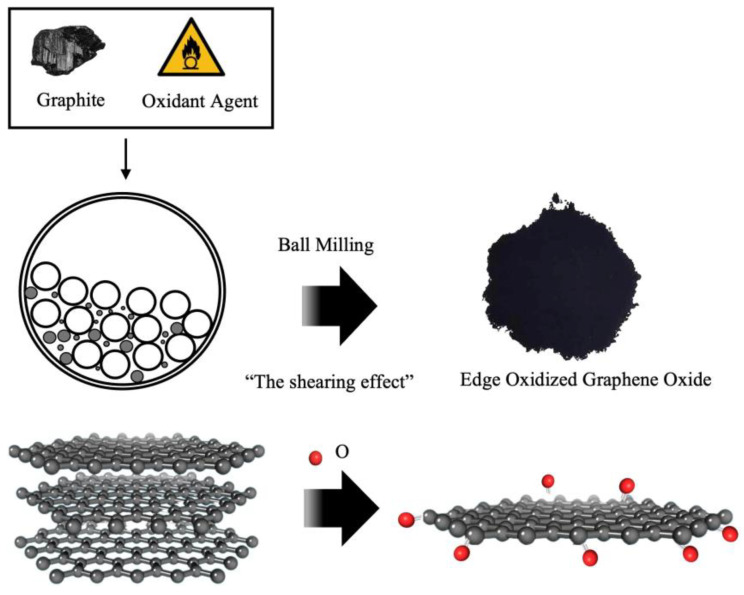
Schematic diagram of the EOGO manufacturing process.

To overcome the cost limitation, a cost-effective graphene oxide, edge-oxidized graphene oxide (EOGO), was introduced. EOGO features oxygen groups located at the edges of graphene, generated through ball milling technology to address cost-related concerns [18]. The detailed process of EOGO production is presented in Figure 1. Relatively inexpensive carbon nanomaterials can be manufactured at less than USD 100 per kilogram using this method. Compared to CNTs, which are often used as additives, which cost USD 400 per kilogram, and GO, which costs USD 300 per gram, EOGO has a significant price advantage.

EOGO has been used in cement and concrete composites as an additive and has shown higher engineering performance [19,20,21]. When EOGO was added into cement and the mechanical properties were measured, it was found that the best effect was achieved with an addition rate of 0.05% [22]. Although there have been many studies on the beneficial effects of EOGO on cement-based materials, there is insufficient information on the application of EOGO to geopolymers. In this study, we investigated the effects of adding a small amount of EOGO to a fly ash geopolymer, a CO_2_ emission-reducing material, on the mechanical properties and durability. The effects of the heat condition and EOGO contents on the development of the mechanical properties in the FA-EOGO geopolymer and FA-EOGO geopolymer were measured and compared. The mechanical properties measured included compressive strength and elastic modulus. The elastic modulus was calculated from the resonance frequency obtained through FFRC, a non-destructive method. Density and porosity were measured to analyze and understand the relationship between voids, strength, and stiffness, and the setting time was measured to investigate the effect of EOGO on the hardening of the geopolymer. Therefore, this study can be a basis for investigating the effects of EOGO on geopolymers before using it as an additive in geopolymers.

## 2. Materials and Specimen Preparation

### 2.1. Materials

The fly ash used in this study was obtained from the combustion of coal at the Dangjin thermal power plant, where the combustion temperature exceeds 900 °C. Table 1 summarizes the chemical composition of fly ash. The composition table of fly ash is from a report provided by a cement company called “Sampyo” in Seoul, South Korea. The ingredients included in the balance are very small in amount, and there are no hazardous ingredients such as heavy metals. This fly ash is classified as Class F fly ash according to ASTM C618 [23], consisting of more than 70% of the sum of SiO_2_, Al_2_O_3_, and Fe_2_O_3_, and less than 10% of CaO.

The EOGO used in this experiment was produced using the mechanochemical method, shown in Figure 1, instead of Hummers’ method. EOGO manufactured in this way contains functional oxygen groups located at the edges. Due to its chemical properties, it has been named edge-oxidized graphene oxide. Compared to typical GOs produced by the conventional Hummers’ method, which contain 4–50% oxygen groups, the oxygen functional group of EOGO is relatively low at 5–10%. However, it has a high surface area of 200 m^2^/g, which makes it highly reactive. Due to its particle size of 450 nm and thin thickness consisting of multiple layers of less than 10 nm, it is effective for pore filling and bridging between particles, as shown in several studies [24,25]; thereby, it is believed that EOGO may enhance the strength of the geopolymer. Table 2 presents the basic properties of EOGO. Table 2 was provided by “Garmor” of Orlando, Florida. The density in Table 2 is the bulk density obtained according to ASTM C188. In EOGO, the oxygen content is 5–10%, and carbon accounts for more than 99.8% of the nonoxygen composition.

**Figure 2:**
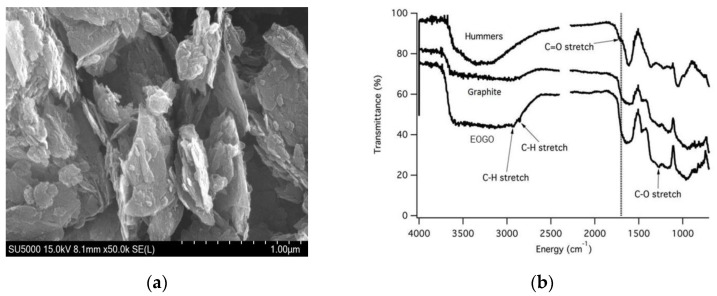
EOGO’s material characterization: (**a**) SEM image of EOGO; (**b**) FTIR analysis of EOGO.

Figure 2a presents a scanning electron microscopy (SEM) image of EOGO, which was obtained from GSEM in Yongin, South Korea. It demonstrating that EOGO has a platelet structure comprising several thin layers. Figure 2b presents the Fourier Transform Infrared Spectroscopy (FTIR) analysis of EOGO, which was obtained from GSEM in Yongin, South Korea. It indicating the functional groups present in EOGO, specifically –OH (hydroxyl) and –COOH (carboxyl). The FTIR results show the differences in the chemical structures of Graphite, GO, and EOGO. Each sample shows different peaks depending on the degree of oxidation. In the case of Graphite, there is no distinct oxygen-related peak because it is almost unoxidized. GO shows distinct oxygen groups at 1000~1700 cm^−1^ due to oxidation treatment. However, in the case of EOGO, since the degree of oxidation is lower than that of GO, a partially separated peak is shown between 1000 and 1700 cm^−1^. EOGO has fewer functional groups than GO produced from Hummers’ method.

Geopolymers are activated in an alkaline environment. In this experiment, the alkali activators used for the alkaline environment were sodium hydroxide pellets, which were mixed with water to produce a sodium hydroxide solution at a concentration of 14 mol. Additionally, a commercial sodium silicate solution (33% SiO_2_, 14% Na_2_O, and 53% H_2_O) was used. The water used in the experiment was tap water.

### 2.2. Mixture Design and Specimen Preparation

The mixture designs used in this experiment are presented in Table 3. There are four groups of fly ash (FA) geopolymer paste with EOGO added at 0%, 0.1%, 0.5%, and 1%. The Water/Solid ratio for all groups is 0.3, and water is included in the alkali activator for all groups.

**Figure 3:**
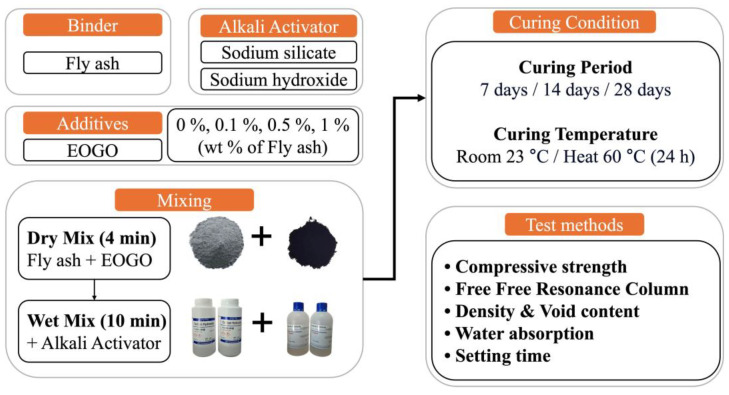
Experimental design and performance tests.

Figure 3 illustrates the experimental design of this study. It is known that FA geopolymers have high compressive strength when cured at elevated temperatures. Therefore, in this study, both room and heat curing were employed. For the heat curing, the specimens were cured at 60 °C for 24 h, and then cured at room temperature for the remaining period. To prevent moisture loss during high-temperature curing, all specimens were cured in zip lock bags. For the performance check, the FA geopolymer was characterized in terms of mechanical, rheological, and durability performance. Curing times of 7, 14, and 28 days were selected for mechanical tests (e.g., compressive strength, FFRC).

## 3. Test Method

### 3.1. Compressive Strength Test

The compressive strength test was conducted according to ASTM C109 [26]. The specimen had a length of 50 mm on one edge, and considering the curing environment, demolding was performed on the second day of curing. Compressive strength tests were conducted at 7, 14, and 28 days, using a Universal Testing Machine (JH Tech in Yongin, South Korea) for the experiments. The load was applied at a constant speed (3 mm/min) to ensure that it was evenly distributed on both the upper and lower surfaces of the specimen. Three specimens were used for each condition, and the average value was applied as the compressive strength.

### 3.2. Free-Free Resonance Column Test

**Figure 4:**
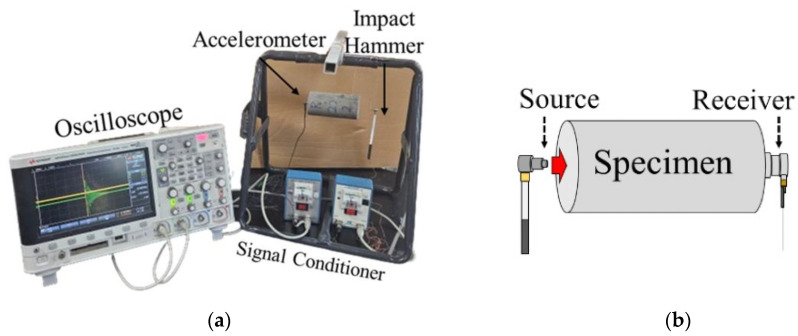
Method of Free-Free Resonance Column test: (**a**) experiment setup; (**b**) impact of the FFRC test.

As shown in Figure 4a, the Free-Free Resonance Column (FFRC) test consists of a hammer and an accelerometer that detects wave signals. The specimens were made in the form of cylinders with a diameter of 50 mm and a height of 100 mm. The experiment was conducted in a state where both ends were free, and the stiffness was obtained through elastic waves generated by external impacts, according to the theory of elastic wave propagation. When the hammer impacts the specimen as seen in Figure 4b, resonance occurs. The resonance frequency, determined via FFT, is used to compute the unconfined compression wave velocity (*V_c_*), and Young’s modulus (*E*) is then determined via Equations (1) through (3):

(1) λ =2L

(2) $$V_{c}=f\times \lambda$$

(3) $$E=\rho \times V_{c}^{2}$$

where λ = wavelength, L = length of the specimen, f = resonance frequency, and ρ = density of the specimen in g/mm^3^.

### 3.3. Density and Void Contents

The density and porosity experiments were conducted according to ASTM C1754 [27]. The specimens were made in the form of cylinders with a diameter of 100 mm and a height of 50 mm, and the experiments were carried out on specimens that had been cured for 28 days. Before the tests, the specimens were dried in an oven at 50 °C, measuring the dry weight at 24-h intervals until the change in weight was less than 0.5%, at which point the experiments were conducted.

### 3.4. Water Absorption Test

The water absorption test was conducted according to ASTM C1585 [28]. For the absorption test, waterproof paint was applied to all sides of the specimen, except for the bottom, which had a diameter of 100 mm and a height of 50 mm. The height of the water surface was maintained at 3 mm from the bottom of the specimen, and the water absorption test was performed. The weight of the absorbed water was continuously measured to calculate the water absorption amount *I* using Equation (4).

(4) $$I=\frac{m_{t}}{a*d}$$

*I* = absorption;

*m_t_* = change in specimen mass in grams, at time t;

*a* = exposed area of the specimen, in mm^2^;

*d* = density of water in g/mm^3^.

### 3.5. Setting Time Test

The initial and final setting times of the geopolymer were determined according to ASTM C191-21 [29]. The mixed geopolymer paste was poured into a mold with a diameter of 70 mm and a height of 40 mm, and the penetration was measured by dropping a Vicat needle every 15 min. The point at which the penetration reached 25 mm was defined as the initial set, and the point where the penetration was 0 mm was defined as the final set.

## 4. Results and Discussion

### 4.1. Compressive Strength Test

**Figure 5:**
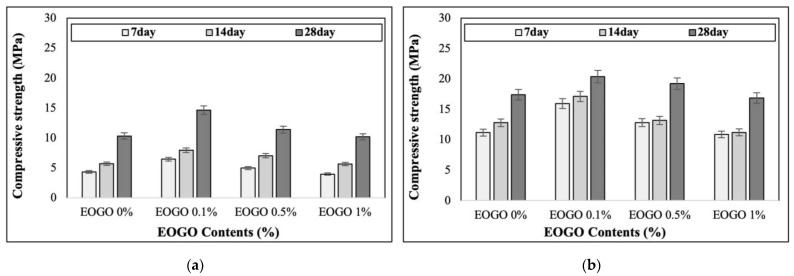
Effect of EOGO content on compressive strength of FA-EOGO geopolymer: (**a**) room curing; (**b**) heat curing (60 °C).

The results of compressive strength are shown in Figure 5. Figure 5a presents the results under room curing conditions, while Figure 5b shows the results under heat curing conditions. The results indicated that the compressive strength increased with longer curing times, irrespective of the EOGO content. This is due to the extended curing time, which allows for further polymerization and enhances long-term strength.

The FA-EOGO geopolymer demonstrates high compressive strength when cured at elevated temperatures. This is because the heat energy applied during heat curing accelerates polymerization, resulting in the formation of additional Si-O-Al bonds and a reduction in unreacted fly ash [30]. High temperatures remove water from the pores, resulting in a denser structure. This can lead to an increase in compressive strength [31].

Under room curing, the 28-day compressive strengths of 0%, 0.1%, 0.5%, and 1% EOGO were 10.33 MPa, 14.64 MPa, 11.38 MPa, and 10.20 MPa, respectively. On the other hand, under heat curing, the 28-day compressive strengths of 0%, 0.1%, 0.5%, and 1% EOGO were 17.39 MPa, 20.40 MPa, 19.22 MPa, and 16.87 MPa, respectively, showing that the highest compressive strength was observed at 0.1% EOGO.

Compared to the control specimen (with no addition of EOGO), 0.1% EOGO increased the strength by 42% under room curing and by 17% under heat curing. The highest compressive strength was observed at 0.1% EOGO. It seems that the uniform dispersion of EOGO results in a “bridging” effect in the paste. However, as the EOGO content increases, EOGO becomes agglomerated due to its electrostatic interactions. The agglomeration results in the formation of large pores, which may reduce the compressive strength of the geopolymer paste.

### 4.2. Free-Free Resonance Column Test

**Figure 6:**
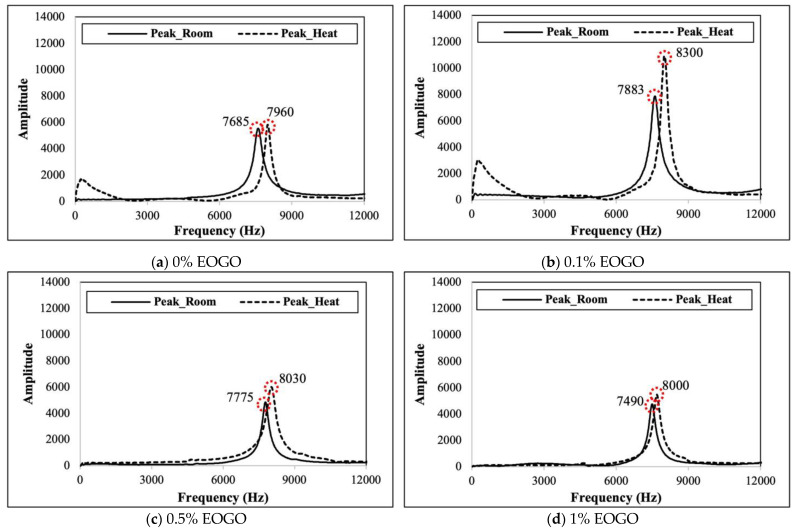
Resonance frequencies of FA-EOGO geopolymers.

The results of the elastic modulus were obtained through the FFRC test. When a hammer hit the specimen, as seen in Figure 4, the resonance frequency was measured. The first modes of the resonance frequency are shown in Figure 6. It is shown that heat curing produced a higher resonant frequency than room curing. At 1% EOGO, the highest resonance frequency was observed, exhibiting 7883 Hz and 8300 Hz for room and heat curing, respectively.

**Figure 7:**
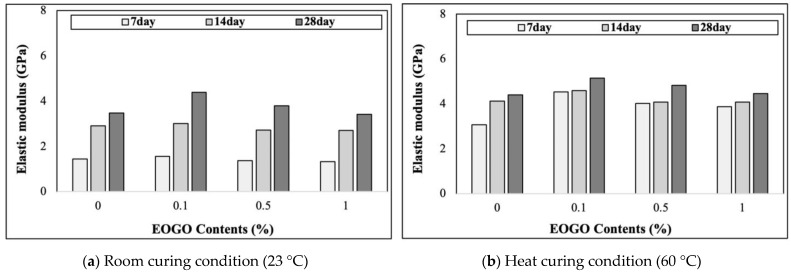
Effect of EOGO content on elastic modulus of FA-EOGO geopolymer: (**a**) room curing condition (23 °C); (**b**) heat curing condition (60 °C).

As seen in Figure 7, under 28-day room curing, the elastic modulus values of 0%, 0.1%, 0.5%, and 1% EOGO are 3.48 GPa, 4.38 GPa, 3.80 GPa, and 3.41 GPa, respectively. On the other hand, under heat curing, the elastic modulus values of 0%, 0.1%, 0.5%, and 1% EOGO are 4.39 GPa, 5.15 GPa, 4.82 GPa, and 4.45 GPa, respectively. The elastic modulus of the geopolymer increases with the curing times. The effect of heat curing is also observed in the elastic modulus of the geopolymer, indicating that a dense structure is formed in the early stages, which results in a high elastic modulus. The elastic modulus according to the EOGO content is at a maximum at 0.1%, and the elastic modulus gradually decreases after 0.1%. These phenomena are similar to compressive strength, suggesting that the agglomeration of EOGO may not fill micropores in the paste, which reduces the density. As a result, the EOGO agglomeration causes lower wave velocity (*V_c_*), resulting in a lower Young modulus (*E*).

### 4.3. Density and Void Content

**Figure 8:**
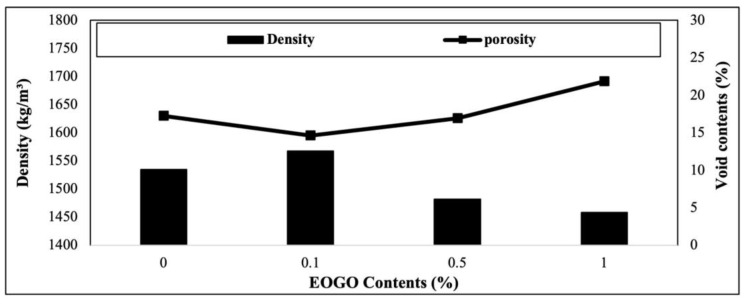
Effect of EOGO content on density and void contents of FA-EOGO geopolymer.

The results of the density and void contents are presented in Figure 8. As the EOGO content increases, the density increases, with the maximum at 0.1% EOGO; however, it decreases after 0.1%. At 0.1% EOGO, the maximum density is 1568 kg/m^3^, and the minimum void content is 15%. The densities of 0.5% and 1% EOGO are lower than the control (0% EOGO). The results of the density and porosity show that EOGO was most uniformly dispersed at 0.1%, suggesting that nano-sized EOGO may fill the micro-sized pores. At 0.5% and 1% EOGO, a higher number of air voids can be formed due to the agglomeration of EOGO, which decreases the density and increases the void content.

### 4.4. Water Absorption Test

**Figure 9:**
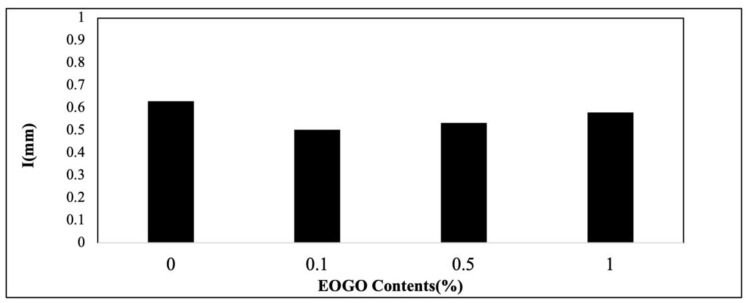
Effect of EOGO content on water absorption of 28-day-cured FA-EOGO geopolymer specimen.

It is known that geopolymers generally have a more porous structure than typical cement/concrete [32]; thus, their water absorption is higher [33]. The results show that the water absorption of the geopolymer ranges from 0.5 to 0.63 mm (see Figure 9). At 0.1% EOGO, the water absorption shows the lowest value of 0.5 mm due to the pore-filling effect. The specimens of 0.5-1% EOGO show slightly lower absorption values than the control specimen. The trend is well matched with the result of the density test (see Figure 8). It is believed that a higher amount of EOGO (e.g., 0.5% or 1%) results in a lower density of the mixture, probably forming a larger size or number of pores.

### 4.5. Setting Time

**Figure 10:**
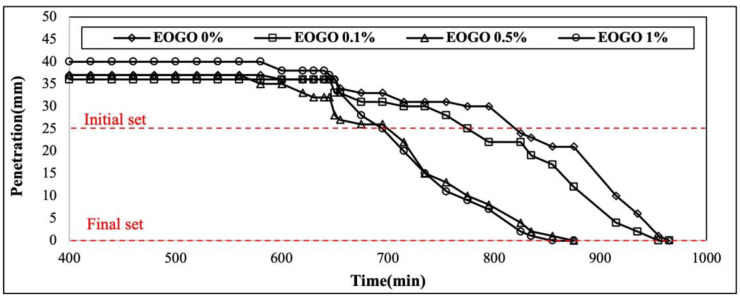
Vicat needle penetration over time for the FA-EOGO geopolymer.

**Figure 11:**
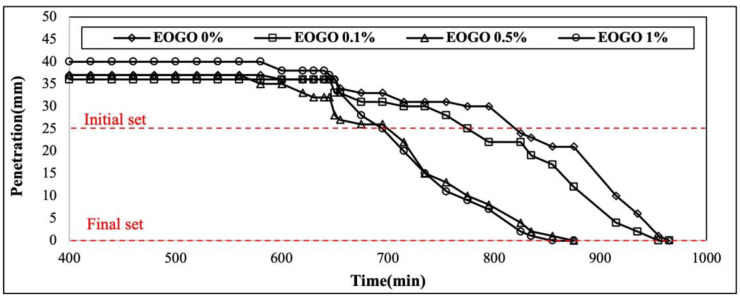
Setting times of the FA-EOGO geopolymer paste.

The results of the setting time test are presented in this section. Figure 10 presents the result of Vicat needle penetration over time, and Figure 11 presents the initial setting time and final setting time according to the addition of EOGO. EOGO at 0.1% exhibits a slight reduction in both initial and final setting times when compared to 0% EOGO.

As seen in Figure 10, 0.5% EOGO and 1% EOGO exhibit a significant decrease in penetration over time, with the most rapid and significant reduction in 1% EOGO. When 1% EOGO and 0% EOGO are compared, the difference in the initial setting time is about 80 min, and the difference in the final setting time is about 100 min. It is confirmed that the addition of EOGO reduces the setting time of the geopolymer (see Figure 11).

## 5. Discussion

In this study, the effect of EOGO on an FA geopolymer was evaluated with multiple mechanical and “durability” tests. The optimum performance was determined at 0.1% EOGO, exhibiting the maximum strength. EOGO may positively affect the geopolymer paste through two functions: (a) a “bridging” effect in the paste and (b) a filling effect in the micropores. According to the studies of Saafi et al. and Qureshi et al., cross-linked GO sheets can be generated by adding very low contents of carbon nanomaterials [23,24]. Through this mechanism, when EOGO is well distributed in the paste, these nanosheets can hold each particle and microcrack under loading, thus leading to higher compressive strength. Additionally, EOGO can fill micropores in the paste, thus causing a higher density and a lower void content, resulting in higher strength. However, as the EOGO content increases, exceeding the optimum content of 0.1% EOGO, agglomeration may occur, and its positive impact is negligible. According to Drabczyk et al., geopolymers have potential as sustainable construction materials due to their low emission of greenhouse gases and low energy demand during production [34]. Additionally, unlike typical GO and rGO, EOGO has a much lower cost (less than USD 100 per kg); thus, its applications to civil engineering are broad and promising. Additionally, since EOGO has a reduced amount of oxygen groups (connected along the edge of the GO sheet), further studies are needed to investigate the flow properties of geopolymers with nanomaterials added, which can be effectively applied in geotechnical applications such as grouting materials for subsurface cavities.

## 6. Conclusions

In this study, EOGO was used as an additive in a fly ash geopolymer. While varying the EOGO content, the effect of EOGO on fly ash geopolymer mixtures was investigated with respect to mechanical and “durability” performance. Two curing conditions, room vs. heat curing, were employed for the FA-EOGO geopolymer specimens. Compressive strength and FFRC tests were conducted to evaluate strength and stiffness, respectively. Subsequently, density, porosity, water absorption, and setting time were measured. The following conclusions have been drawn:

- Through compressive strength and FFRC tests, it was found that heat curing led to a higher strength and elastic modulus than room curing in all cases. The testing results show that 0.1% EOGO exhibited the maximum strength and modulus. Under 28-day curing, 0.1% EOGO under room curing showed 42% higher strength and 26% higher elastic modulus than the control (no EOGO). On the other hand, 28-day heat curing showed a 17% increase for both strength and modulus.
- It was expected that when EOGO was added to the fly ash geopolymer, the nano-sized EOGO would fill the pores of the geopolymer, and the effect would be best observed at 0.1% EOGO. However, 0.5% and 1% EOGO showed a lower density and higher void contents compared to 0.1% EOGO. This is probably due to the agglomeration of EOGO.
- Due to the pores and high surface area of EOGO, 0.5% and 1% EOGO showed higher water absorption and a decreased setting time. Because EOGO filled the pores, but agglomeration occurred with a higher amount of EOGO, more pores were created and water was absorbed through these pores. And the setting time was decreased because the high surface area of EOGO reduced the free water.

In conclusion, the mechanical properties of fly ash geopolymers can be increased by adding EOGO, which is effective even at a smaller amount than nanosilica and nanoclay, and is much cheaper than GO. Therefore, EOGO has potential as an additive to geopolymers, and FA-EOGO geopolymers can be a good alternative to traditional cementitious materials since they can reduce carbon emissions [11,35,36].

## Figures and Tables

**Table 1:** Composition of fly ash.

SiO_2_	Al_2_O_3_	Fe_2_O_3_	CaO	LOI	Balance
57.3	27.15	8.2	3.7	2.39	1.26

**Table 2:** Properties of EOGO.

**Oxygen** **(%)**	**Nonoxygen** **Composition**					
	**Carbon** **(%)**	**Specific Gravity**	**Surface Area** **(m^2^/g)**	**Mean** **Particle Size** **(nm)**	**Thickness** **(nm)**	**Density** **(g/cm^3^)**
5–10	>99.8	1.91	200–300	450	~10	1.0

**Table 3:** Mixture designs of FAGO geopolymers.

Mix	Fly Ash (g)	Activator (g)	EOGO (g)	W/S (%)
FA-EOGO-0%	1376	880.6	0	30
FA-EOGO-0.1%	1376	880.6	1.4	30
FA-EOGO-0.5%	1376	880.6	6.9	30
FA-EOGO-1%	1376	880.6	13.8	30

## Data Availability

The original contributions presented in this study are included in the article. Further inquiries can be directed to the corresponding author.
